# Community-based lifestyle intervention for diabetes (Co-LID study) management in rural Nepal: study protocol for a clustered randomized controlled trial

**DOI:** 10.1186/s13063-023-07451-5

**Published:** 2023-07-05

**Authors:** Lal Rawal, Padam Dahal, Grish Paudel, Tuhin Biswas, Rabina Shrestha, Deepa Makaju, Abha Shrestha, Uday Yadav, Berhe W Sahle, Hanako Iwashita, Gaku Masuda, Andre Renzaho, Prabin Shakya, Archana Shrestha, Biraj Karmacharya, Haruka Sakamoto, Rajendra Koju, Tomohiko Sugishita

**Affiliations:** 1 School of Health, Medical and Applied Sciences, Central Queensland University, Sydney Campus, 400 Kent Street, Sydney, NSW 2000 Australia; 2 Appleton Institute, Physical Activity Research Group, Central Queensland University, Rockhampton, Australia; 3 Translational Health Research Institute, Western Sydney University, Sydney, Australia; 4 Science and Math Program, Asian University for Women, Chattogram, Bangladesh; 5 Research and Development Division, Dhulikhel Hospital Kathmandu University Hospital, Dhulikhel, Nepal; 6 Department of public health and community programs, Kathmandu University of Medical Sciences, Dhulikhel, Nepal; 7 National Centre for Aboriginal and Torres Strait Islander Wellbeing Research, The National Centre for Epidemiology and Population Health, ACT, The Australian National University, Canberra, Australia; 8 Centre for Primary Health Care and Equity, University of New South Wales, Sydney, Australia; 9 School of Nursing and Midwifery, Faculty of Health, Deakin University, 221 Burwood Highway, Burwood, Melbourne, VIC 3125 Australia; 10 Melbourne School of Population and Global Health, The University of Melbourne, Melbourne, VIC Australia; 11 Section of Global Health, Division of Public Health, Department of Hygiene and Public Health, Tokyo Women’s Medical University, Tokyo, Japan; 12 School of Medicine, Western Sydney University, Sydney, Australia; 13 Department of Internal Medicine/Cardiology, Kathmandu University of Medical Sciences, Dhulikhel, Nepal

**Keywords:** Type 2 diabetes mellitus, Lifestyle intervention, Health behaviour, Community-based, Cluster randomized controlled trial

## Abstract

**Background:**

Type 2 diabetes mellitus (T2DM) has increased globally; with a disproportionate burden in South and Southeast Asian countries, including Nepal. There is an urgent need for clinically and cost-effective culturally adapted T2DM management programs. In this study, we aim to assess the effectiveness of community based culturally appropriate lifestyle intervention in improving the management and care of people with T2DM.

**Methods:**

We will conduct a cluster randomized control trial to evaluate the effectiveness of community based culturally appropriate lifestyle intervention in improving T2DM outcomes. The trial will be conducted in 30 randomly selected healthcare facilities from two purposively selected districts (Kavrepalanchowk and Nuwakot districts) of Bagmati province, Nepal. The selected healthcare facilities are being randomized into 15 interventions (*n* = 15) and usual care (*n* = 15) groups. Those in the intervention will receive group-based 12 an hour-long fortnightly session delivered over 6 months period. The intervention package includes 12 planned modules related to diabetes care, ongoing support, supervision and monitoring, follow-up from the trained community health workers, and educational materials on diabetes self-management. The participants in the usual care groups will receive pictorial brochure on diabetes management and they will continue receiving the usual care available from the local health facilities. The primary outcome is HbA1c level, and the secondary outcomes include quality of life, health care utilization, and practice of self-care behaviour, depression, oral health quality of life, and economic assessment of the intervention. Two points measurements will be collected by the trained research assistants at baseline and at the end of the intervention.

**Discussion:**

This study will provide tested approaches for culturally adapting T2DM interventions in the Nepalese context. The findings will also have practice and policy implications for T2DM prevention and management in Nepal.

**Trial registration:**

Australia and New Zealand Clinical Trial Registry (ACTRN12621000531819). Registered on May 6, 2021.

**Supplementary Information:**

The online version contains supplementary material available at 10.1186/s13063-023-07451-5.

## Introduction

Globally, type 2 diabetes mellitus (T2DM) accounts for 90–95% of all cases of diabetes and contributes to around 3% of total disability-adjusted life years (DALYs) [1–3]. The International Diabetes Federation (IDF) reported that 537 million adults were living with diabetes in 2021 and projected to reach 784 million by 2045 [4]; with global expenses around US$ 966 billion in 2021 and are projected to reach US$ 1054 billion by 2045 [4]. The IDF’s estimates indicate that more than 75% of people with diabetes live in low and middle-income countries (LMICs) [4]. Recent data suggest that the larger proportion of people with T2DM resides in South and Southeast Asian countries [4, 5]. In 2021, around 90 million adults (aged 20–79 years) were living with diabetes in the Southeast Asia region and 51.2% remained undiagnosed [4, 6]. T2DM imposes significant economic burden on patients, their families, health systems, and national economies, both directly due to treatment costs or indirectly because of loss of productivity [7].

Over the years, Nepal has been experiencing an increased burden of noncommunicable diseases (NCDs) including T2DM [8]. The prevalence of T2DM among adults in Nepal is estimated to be 8.5%, and a further 5.7% had raised blood glucose levels. A 2020 systematic review reported a pooled diabetes prevalence of 8.5% in Nepal [9]. Furthermore, a WHO Stepwise Surveillance (STEPS) survey reported 5.8% of adult Nepalese population in 2019 had raised blood glucose [10]. T2DM has represented a significant financial burden for Nepal, resulting in US$ 115.8 million in total diabetes-related health expenditure in 2021, and projected to reach US$ 190.5 million by 2045 [4]. Evidence also shows that the prevalence of T2DM risk factors is very high. For example, in 2019, the WHO STEP survey reported that around 24% of Nepalese were overweight and had raised blood pressure (BP), 17% had smoking behaviour, 11% had high cholesterol levels, 8% had insufficient levels of physical activity, 7% people consumed alcohol (highest 12% in the male), and 97% participants did not consume sufficient fruits and vegetables [10]. This suggests that the number of T2DM cases will continue to grow in the coming years.

Inadequate access to and use of healthcare services and high out-of-pocket expenses for management and care can increase the likelihood of T2DM complications, which in turns become a significant burden for the individuals and healthcare system. Therefore, there is an urgent need for developing clinically and cost-effective culturally appropriate health behaviour interventions to improve T2DM outcomes. In this study, we aim to assess the effectiveness of community based culturally appropriate lifestyle intervention in improving the management and care of people with T2DM.

## Methods

### Trial design

This study follows prospective, single-blinded end-point assessment of a 2-arm randomized controlled trial (RCT) with blinding of the statistician in order to perform cluster randomization. The intervention time is 6 months with assessments of outcomes at baseline and at the end of the intervention. This protocol was designed in accordance with the Standard Protocol Items: Recommendations for Interventional Trials (SPIRIT) Statement (Cite) (Additional file 1) [11].

### Study setting

**Fig. 1:**
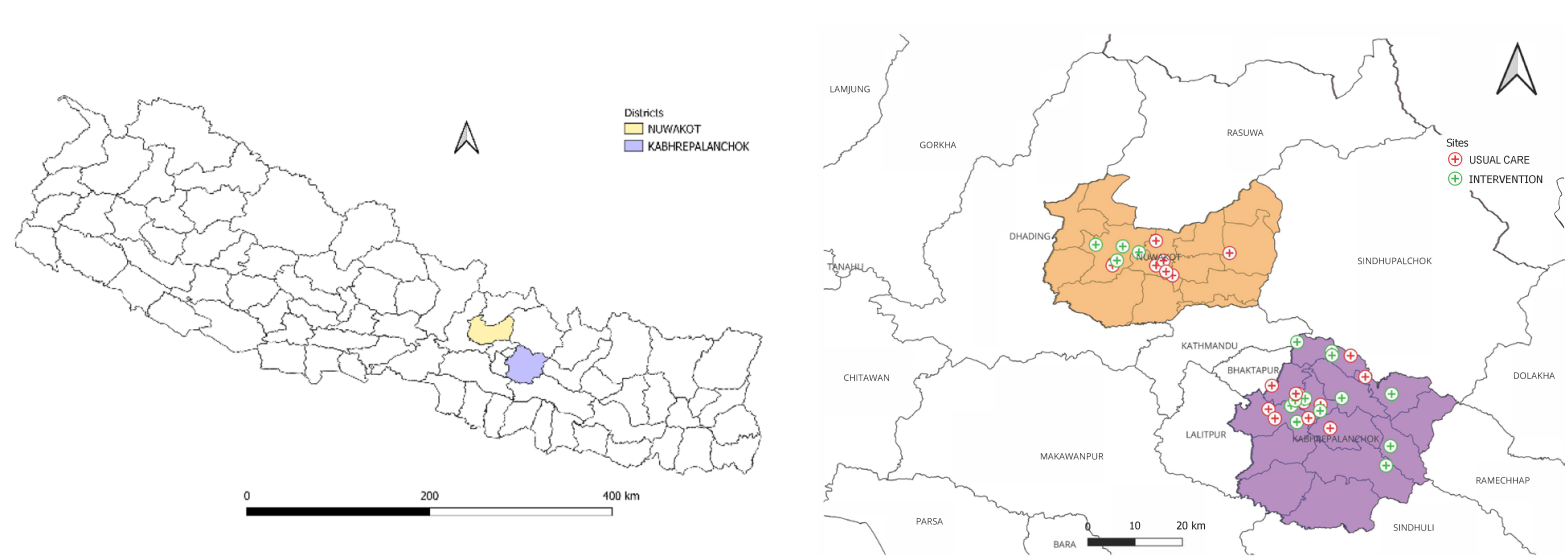
Map of Kavrepalanchowk and Nuwakot districts with selected study clusters/health facilities

This community-based lifestyle intervention for diabetes (Co-LID study) management is being implemented in two selected districts (Kavrepalanchowk and Nuwakot districts) of Bagmati province, Nepal (Fig. 1). The total population of Kavrepalanchowk district is 366,879 [12], and there are altogether 172 health facilities (132 public and 40 private) within the district [13]. Similarly, Nuwakot district comprises 262,981 people [12] with 97 healthcare facilities (83 public and 14 private) within the district [13]. In this study, the health centres such as health posts, primary health care centres, urban health centres, community health units, and the outreach centres of Dhulikhel hospital in Kavrepalanchowk and Nuwakot district are being considered as the clusters. The baseline and endline data will be collected from the participants from all clusters (both intervention and usual care arms).

### Study population

The target participants of this interventional study are those clinically diagnosed with T2DM and aged 30–70 years who are capable for coping with active lifestyle management. Those who have clinically diagnosed type 1 diabetes mellitus, currently pregnant, below the age of 30 years, and with mental illness were excluded in this study. The interventional group will receive the allocated interventional packages in the local healthcare facility as scheduled.

### Clusters

**Fig. 2:**
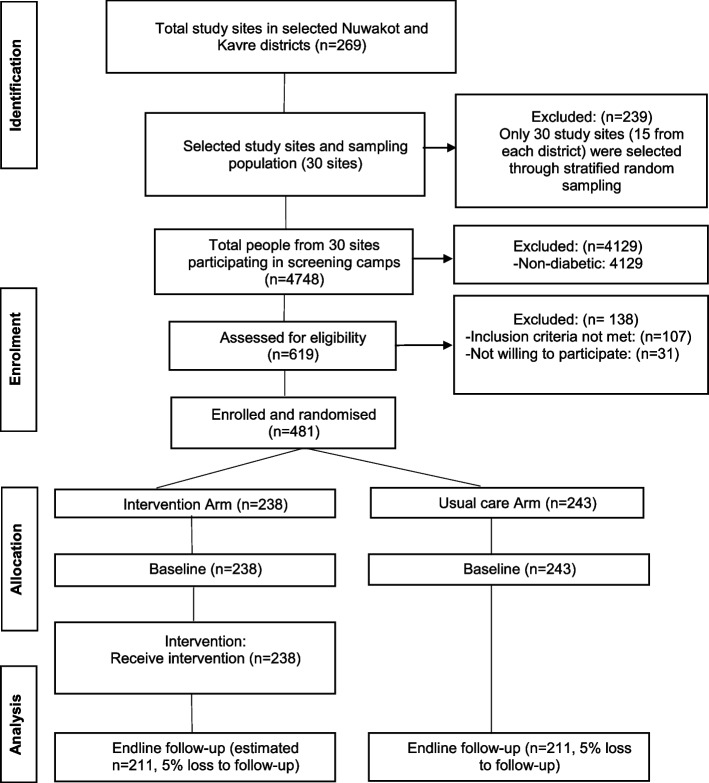
CONSORT diagram of the community-based T2DM intervention in Nepal

Unit of cluster for this study are healthcare facilities within the districts with at least 12 people in each cluster who are clinically diagnosed with T2DM. In total, 30 clusters from both districts are being selected adopting the stratified random sampling. The selected clusters will be randomly allocated as intervention and usual care groups in 1:1 ratio. To avoid the inter-cluster contamination, the nearby/neighbouring clusters are allocated as usual care and intervention groups. The interventions will be evaluated with two cross-sectional surveys, i.e. at baseline and in endline after the 6 months of interventions (Fig. 2).

### Randomization

The participants meeting the inclusion criteria and able to provide the consent for their participation in the study will be randomly allocated to intervention and usual care arms. A total of 30 clusters that met the inclusion criteria (i.e. 20 health centres from Kavrepalanchowk and 10 from Nuwakot districts) are being randomly allocated into 15 interventional clusters and 15 usual care clusters by the statistician not involved in this study.

### Sampling

Healthcare facilities, primarily the ones run by the public sector, have been considered as study sites. There are a total of 269 public and private healthcare facilities (i.e. 172 in Kavrepalanchowk and 97 in Nuwakot) in these selected districts of Nepal [13]. Stratified random sampling technique was adopted to enrol the 30 study sites (15 each from Kavrepalanchowk and Nuwakot district) if the health centres meet the criteria of 12 type 2 diabetes participants that could be enrolled in our study. With this, we will use Health Management and Information Systems (HMIS) registries of selected healthcare facilities to identify the people clinically diagnosed with T2DM. Given the record keeping of NCDs including diabetes in HMIS registries is not mandatory in public health care facilities, we also organized the diabetes screening camps in those places to identify the people with T2DM. All patients clinically diagnosed with T2DM and those diagnosed during the screening camps were enrolled in the study. Those diagnosed with T2DM in the screening camps were sent to nearest health facility and pathology for confirmatory test before they were enrolled in the study. At least 12 people clinically diagnosed with T2DM were required in each cluster. The clusters were then randomly allocated into intervention or usual care arms (Fig. 2).

### Sample size

The aim of this study is to assess the effectiveness of intervention in terms of reduction of mean HbA1c levels from 7.5% at baseline to 7.0% at 6 months follow-up among the intervention participants compared with estimated no or minimal changes in participants of usual care arm (HbA1c levels 7.5%), with at least 80% power and 95% confidence interval (CI) and assuming an intra-class correlation coefficient (ICC) of 0.050 and estimated HbA1c mean standard deviation (SD) of 1.3, as reported in studies that used diabetes self- management programs in the past [14]. The cluster RCTs implemented in similar settings of the Asian subcontinent in the past have used ICC of 0.02 [15] to 0.05 [16]. Allowing for cluster randomization with 15 clusters each arm, a minimum of 12 individuals would be required per cluster, equating to a total sample size of 180 per arm at 6 months follow-up. With the adjustment of possible maximum 15% attrition rate at 6-month follow-up, we required 211 participants in each arm at the baseline.

#### Recruitment of community health workers (CHWs)

Community health workers are the peripheral frontline health workers of the government health facilities (Primary Health Centre, Health Post, Urban Health Centre) and outreach centres of Dhulikhel Hospital. Any two CHWs (Auxiliary Nurse Mid-wife, Auxiliary Health Worker or Health Assistant) from each of selected health facilities willing to participate in this intervention are enrolled and the consent from their respective health facility in-charge was sought to get them involved in the study. The selected CHWs are trained in terms of health behaviour, patient empowerment, social and emotional support, and overall T2DM self-management by diabetologist, nurse practitioner, physician and oral health specialist, etc.

#### Recruitment of peer supporters

Peer supporters are those individuals who have experiential knowledge of a specific behaviour or stressor and similar characteristics as the target population [17]. Those meeting the eligibility criteria for this study and expressed interest to self-manage lifestyle themselves and help others are identified and invited to act as peer supporters for the study. Those who accept to act as peer supporter are selected and trained to closely work with the trained community health workers (from the respective study site) and provide necessary support to organize the group-based sessions of the intervention such as invitation to sessions, talking to participants, social and emotional support, and encourage to adhere to healthy lifestyles by setting an example. Two peer supporters are recruited from each of the clusters in an interventional arm.

### Interventions

The community-based intervention comprises of combination of intensive training sessions on diabetes self-management programs that include dietary measures, stress management/problem solving, ways to stay away from triggers to drink alcohol and smoke, diabetes medications, diabetes literacy, monitoring blood sugar level, physical activity, health care utilization, oral health, foot care, quality of life, and social and emotional support. These are the group-based (group consists of all participants of a particular cluster of an intervention arm) fortnightly 1-h long counselling sessions to be conducted by trained CHWs. We have altogether 12 modules that are to be conducted fortnightly for 6 months. During the counselling sessions, we will fill the gaps in the participant’s knowledge and empower participants to take the decision to change their lifestyle to prevent complications associated with diabetes. We will have empathetic listening if participants want to share their queries and experiences. In addition, a pictorial book on diabetes management and prevention of complication in Nepali language with the same content as delivered in the group-based counselling sessions will be provided to the study participants. Apart from this, an automated phone call and text messages will be delivered to the participants to remind them of the upcoming fortnightly training sessions. See Additional file 2 for summary intervention modules (Additional file 2).

Moreover, a trained CHW will conduct a fortnightly telephone call session for the first 3 months and then the monthly calls for the rest of the months to ensure adoption and maintenance of healthy self-care behaviours (i.e. healthy eating, physical activities, blood sugar monitoring, taking medicine, foot care, acute and chronic complications awareness, healthy coping, problem solving and reduction of complications) among the study participants [18]. Additionally, online messages (pictorial and audio visual) on lifestyle intervention using mobile phones and smartphones will be delivered to the interventional group over a period of 6 months.

### Usual care

Usual care group will receive standard diabetes management and care provided in accordance with usual care in the selected location. In addition, a pictorial book on diabetes prevention education in Nepali language will be provided in order not to deprive them from awareness raising.

### Outcome measures

The primary outcome measure of this study are glycated haemoglobin levels as defined by the WHO [19]. Secondary outcome measures include quality of life, health care utilization, and practice of self-care behaviour and economic assessment of health behaviour interventions. The primary and secondary outcome measures are briefly presented in Table 1.

**Table 1:** Summary of outcome variables

**Outcomes**		**Measurement tool**
***Primary outcome***		
Glycated haemoglobin (HbA1c) levels		Blood
***Secondary outcomes***		
Quality of life	General	SF-8 [20], EQ-5D [21]
	Diabetes and oral health	Oral Health Impact Profile (OHIP-14) [22]
	Diabetes distress	Perceived Stress Scale (PSS) [23]
	Depression	PHQ-9 [24]
	Blood pressure	NDHS STEPS survey [14]
	Body mass index (BMI)	NDHS STEPS survey [14]
Program effectiveness (process evaluation)		Training for community health workers using Training of Trainers (TOT) modality for local capacity building, training for peer supporters focusing on facilitation and communication skills, monthly peer support meeting, follow-up
Healthcare utilization		Access to healthcare services Visit to health facility in last 6 months Specialist visits in last 6 months
Practice of self-care behaviours		24-h dietary recall and fruits and vegetable intake in a week adapted from SDSCA [15], NDHS STEPS survey for physical activity, MMAS-8 [16] for medication intake
Economic evaluation	Cost-estimation	No. of visits past 6 months to diabetes clinician/endocrinologist No. of visits past 6 months to other clinicians No. of visits past 6 months for emergency/acute care No. of overnight stays past 6 months in hospital (related to your diabetes)
	Cost-effectiveness analysis	Cost of healthcare resource use and EQ-5D
	Long-term economic impact analysis	Markov modelling

### Data collection

Data will be collected through face-to-face interview, at baseline and 6 months after enrolment (Table 2). Research assistants with the health and medical sciences background including lab assistants will be recruited and trained for a week on rapport building, research methods and debriefing of the survey tools. In order to ensure participants retention and follow-up, we will continue working closely with the community health workers, maintain regular follow-up phone calls with the participants, and engage peer supporters in respective study sites. A pre-testing of the survey questionnaires was conducted in the homogeneous non-sampling area and was revised as required. The final set of survey questionnaire was uploaded in the Kobotoolbox/ODK platform and data were collected using the ODK collect app in android tablets.

**Table 2:**
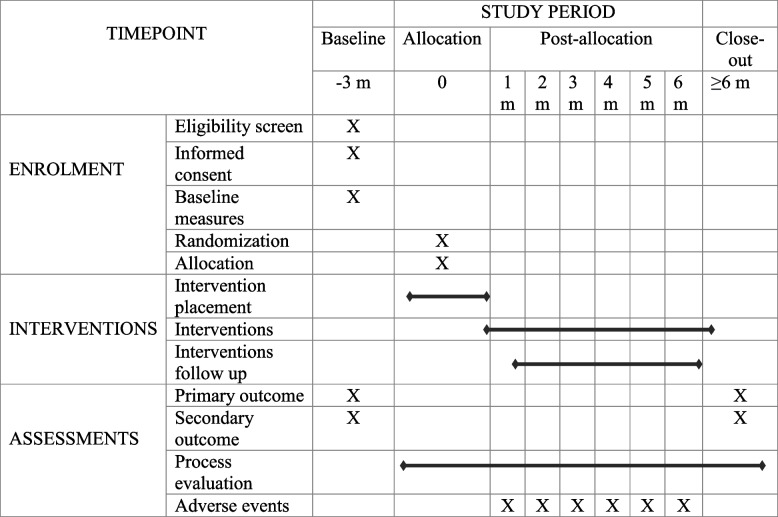
Schedule for enrolment, baseline, intervention, and assessments

*m*, month

Study participants from each cluster will be invited to their local health facility for the data collection. A verbal and written informed consent will be obtained before the start of the interview. This was then followed by structured behavioural interview, anthropometric measurement, and lab test (blood glucose test). Data were collected by using different validated measurement tools based on the study outcomes that are presented in Table 1.

The random blood glucose level will be measured by glucometer at the baseline (HbA1c was taken by a sensitive POCT device by a trained Lab Assistant), and height and weight of the participants will be measured using the measurement tape and weighing machine respectively as part of anthropometric measurement. However, blood will not be stored for the future use in the ancillary studies. Weight will be assessed in the light clothing and barefoot to get the right value. Based on the height and the weight, the body mass index will be calculated dividing the weight (in kilogrammes) by height (in square metres). Waist circumference will also be taken using the STEPs guideline. Blood pressure was measured using appropriate measures for 3 times, the average of which was considered the final blood pressure. A set of validated standard tools was adopted to assess the secondary outcomes such as quality of life, healthcare utilization, and practice of self-care behaviours. Same process will be applied at the endline for the final data collection (Table 2).

### Safety and adherence considerations

The study participants enrolled in both the intervention and usual care arms will be briefed about the study, study purpose and their voluntary participation. A verbal and written consent form will be obtained from the study participants. The confidentiality of the participants will be maintained throughout the study. The proposed study is expected to cause no harm or effect to the study participants, however, if arises will be assessed by the project team members and determine whether any medical attention is required. Any complaints or feedback from the study participants will be recorded throughout the trial. In case of any serious complications raised during the trial, the participants will be seen by the doctor and can be asked to discontinue the trial if needed. The technical advisory committee (TAC) with the representation from the Ministry of Health and Population Studies, Nepal Health Research Council, World Health Organization Nepal, and Universities and Research Institutes in Nepal has been formed and responsible for overseeing the project implementation. This committee also function as trial steering committee (TSC). Kathmandu University, Nepal, is a project implementing organization which will be responsible for identifying potential participants, recruit them and obtain informed consents at the community and local health facility level. Furthermore, we have formed a Stakeholder and Public Involvement Group (SPIG) with the representation of different stakeholders and beneficiaries at community level. These involve the following: local municipal council, district education office, district health office, local health centre, female community health volunteer, mothers’ group, local sport clubs, and representative of consumer groups. We will closely work with the SPIG in order to ensure implementing the project activities smoothly at the community level. The research assistants with the health science background have been trained are being used for the data collection. Participants will be informed about the blood glucose test and the results of the test will be provided to the respective participants. Given this is a small community-based intervention trial and is not expected to involve any high risk or harm to the participants, the data monitoring committee (DMC) has not been established. For auditing, project management group will meet in every fortnightly and the ethical committee from Nepal Health Research Council (NHRC) will monitor in every 6 months. In the event of protocol amendments when required, the principal investigator will notify sponsor and funder and make necessary changes and updates to the trial registry and inform relevant ethical committees.

### Data analysis

#### Intention-to-treat analysis

The data analyses will follow according to the primary and secondary outcome measures between the intervention and usual care arms and at the end of the intervention against the baseline measures. Descriptive statistics will be calculated using mean (± SD) for the continuous variable and frequencies and percentages for the categorical variables. The primary outcome measure (mean HbA1c levels) at the end of 6-month intervention period will be compared between the intervention and usual care arms using generalized linear mixed models with a random cluster effect and adjusting for important baseline confounders. Analyses will follow an intention-to-treat approach including all clusters and participants in the group they are randomized to, regardless of whether they receive or complete the intervention.

Analyses of secondary outcomes will compare the proportion of participants who are adherent to lifestyle change (such as achieved moderate or vigorous physical activity, quit smoking, healthy diet, health care utilization, medication adherence, improved diabetes distress levels) at the end of the 6-month intervention period between the two intervention and usual care groups using generalized linear mixed models with a random cluster effect and adjusting for potential confounders. Variables proposed to interact with intervention will be tested (at *p* < 0.05) by including a cross product of each variable by the intervention in a model together with the main effects. A subgroup analysis will be performed by age, sex, SES, and residential status if there is any statistically significant interaction with the intervention, and the inferential analysis will follow cluster adjustment. In addition, the multiple imputation will be applied to handle the missing data.

An assessment whether any difference in outcome measures between usual care and intervention arms varies by HbA1c levels will be assessed using multi-variate analyses. The significance of subgroup effects will be assessed by tests of interactions of covariates and the intervention effect. We will also assess the differences in outcome measures by subgroups such as broadly classified as low, medium, or high intervention effect.

#### Economic evaluation

Cost, cost-effectiveness and returns of investment analysis will be conducted in the healthcare and societal perspective. Cost of the healthcare resource use will be collected from the study participants and cost of implementing the interventions will be collected prospectively from the project accounts by using the standardize Excel sheet design especially for this project. Incremental cost-effectiveness ratios (ICERs) per quality-of-life year (QALY) gained and returned on investment (ROI) ratio will be calculated, and the robustness of the results will be assessed through sensitivity analysis. In addition, Markov model will be applied to forecast the long-term economic impact of the health behaviour intervention.

#### Process evaluation

To assess the adherence to the intervention protocol, registry of educational sessions and other proposed project activities will be maintained. In addition, briefing and debriefing on project implementation and the potential field level challenges that may arise will be discussed in a regular fortnightly meeting. Mixed method approach will be applied for the process evaluation of the intervention. The research team members will conduct focus group discussion among the study participants, key informant interviews (four from each district) with the community health workers, and peer supporters until we reach the saturation as part of the process evaluation. In addition, four key informant interviews will be conducted with the district health office.

### Dissemination

The findings will be produced in different forms including manuals, intervention guidelines, protocols, scientific publications, workshop, stakeholder meetings, and policy briefing. Findings will also be shared at national and international forums and published in journals audiences will include academic audiences, policy makers, and local health service providers at the end of the project through regional and national forums. Furthermore, the findings will be published in an open-access peer reviewed academic journal.

## Discussion

In this protocol, we have discussed the development, implementation, and evaluation of the community-based behavioural intervention for diabetes management to be implemented in Nepal. To our knowledge, this is the first interventional study aiming to determine the effectiveness of a culturally appropriate health behaviour intervention (using combination of community health workers, peer supporters, and regular telephone calls) in improving management and care of people with type 2 diabetes mellitus in the selected districts of Nepal. The findings of this interventional study are expected to have policy implications in the design and implementation of diabetes management programs in Nepal and similar settings.

Nepal in recent years is facing increasing problem of non-communicable diseases (NCDs) including diabetes [25, 26]. According to the World Health Organization (WHO), estimated 60% of total deaths (age between 30 and 70 years) are attributable to NCDs and related conditions in Nepal [27]. NCDs attributable deaths have been significantly increased over the years, from 66% in 2016 [28] to 71.1% in 2019 [29]. In Nepal, 61.2% of total DALYs were due to NCDs, followed by communicable, maternal, neonatal, and nutritional (CMNN) diseases (29.3%) and injuries (9.6%) in 2019 [29]. A hospital-based cross-sectional survey showed overall prevalence of NCDs as 31%, with chronic obstructive pulmonary disease (43%), followed by cardiovascular disease (40%), diabetes mellitus (12%), and cancer (5%). The majority of CVD cases were hypertension (47%) followed by cerebrovascular accident (16%), congestive cardiac failure (11%), ischemic heart disease (7%), rheumatic heart disease (5%), and myocardial infarction (2%) [30]. A systematic review by Mishra et al. showed that the prevalence of hypertension ranged from 22.4 to 38.6%, diabetes from 4.1 to 9.5%, CVD 5.7%, and any form of mental illness 37.5%, and each year, 8000 to 10,000 people are diagnosed with any form of cancer in Nepal, with lung cancer as the main form of cancer [31]. Other studies also reported high prevalence of NCDs in Nepal, including hypertension (34%) [32] and diabetes (15%) [33]. Furthermore, chronic obstructive pulmonary diseases (43%) were the most common NCDs among outpatients followed by cardiovascular disease (40%), diabetes mellitus (12%), and cancer (5%) [30].

While the burden of NCDs including diabetes in Nepal is increasing, the government of Nepal has developed a number of NCD-related policies, and the programs/services are being implemented. However, the implementation aspect of the policies remains weak [34]. The PEN Package in Nepal has been implemented since 2013/2014; however, there has been a need for strengthening the ongoing implementation aspects of the PEN package at the primary care level health facilities. The lack of screening and awareness at the community level implies the greater need of people centred NCDs interventions. This requires development and implementation of comprehensive self-management program to improve the health outcomes of the people living with NCDs [35]. There has also been a need for developing and implementing the life-course approach which could include secondary and tertiary preventive approaches to maintain their NCDs conditions and reduce the potential complications due to uncontrolled NCDs [36]. While Nepal is committed to offer universal access to essential health services for NCDs, no options to supplement the existing primary healthcare delivery system has been identified. Evidence based on capacity building of CHWs, the process to deliver interventions through CHWs, and the effective implementation of community based CHW-led intervention are essential.

The findings of this study are expected to support in developing capacity of health professional and researcher involved in chronic disease management in Nepal. Additionally, the outcomes of the study can also prove the effectiveness of the lifestyle modification related programs that can be applied in future in diabetes or similar kind of disease management approach in integration with other NCDs that benefit from this approach. The findings will also provide evidence base to scale up the cost-effective diabetes management programs to the National level. The findings of this study will have policy and services delivery levels implications in the design and implementation of cost-effective, culturally appropriate, context specific community-based approaches to manage NCDs including diabetes in Nepal.

This study has few important limitations. The study designed to implement only two selected districts of Nepal which may limit the generalizability of the findings obtained from this study. Social desirability of bias may arise while conducting qualitative interviews. Possible effect due to cross contamination may arise when the participants migrate from one study location to another location without informing the project team members. Despite these possible limitations, we will endeavour to effectively and per protocol implement the project activities.

## Trial status

The recruitment of the participants has completed, and the data collection is in progress. The protocol version number is 2.0 (October 2021). The primary reason for the amendment was COVID-19 pandemic. Due to the delay in field data collection and limited timeframe of the project funding, the duration of the intervention was revised from 12 months to 6 months. However, the total number of modules of the intervention remained unchanged (12 modules as originally planned). We are aiming to conduct endline data collection around mid-2023.

## Supplementary Information

**Additional file 1.** SPIRIT checklist.

**Additional file 2.** Summary intervention module for management of diabetes.

## Data Availability

The datasets analysed during the current study and statistical code are available from the corresponding author on reasonable request. Furthermore, the participant’s information materials and informed consent form are available from the corresponding author on request.

## Abbreviations

BMI: Body mass index
CHW: Community health worker
CoLID: Community-based lifestyle intervention for diabetes
COPD: Chronic obstructive pulmonary disease
CVD: Cardiovascular disease
FCHVs: Female community health volunteers
IDF: International Diabetes Federation
NCDs: Non-communicable diseases
PEN: Package of Essential Non-Communicable
RCT: Randomized control trials
Re-AIM: Reach, Effectiveness, Adoption, Implementation and Maintenance
T2DM: Type 2 diabetes mellitus
WHO: World Health Organization
