# Supplementary material for: Community-based lifestyle intervention for diabetes (Co-LID study) management in rural Nepal: study protocol for a clustered randomized controlled trial
Source: Trials. 2023 Jul 5;24:441. doi: 10.1186/s13063-023-07451-5 (PMC10320880; doi:10.1186/s13063-023-07451-5)
Supplement: Supplementary file 2 — Additional file 2. Summary intervention module for management of diabetes. [file 13063_2023_7451_MOESM2_ESM.docx]

**Appendix A: Summary intervention module for management of diabetes**

| **Module 1** | Introduction of diabetes, risk factors, symptoms and investigation of diabetes. Detecting hypoglycaemia, complication of diabetes mellitus |
| --- | --- |
| **Module 2** | Blood pressure, hypertension and diabetes |
| **Module 3** | Utilization of health services for diabetes management |
| **Module 4** | Physical activity and management of diabetes |
| **Module 5** | Depression, stress and time management |
| **Module 6** | Healthy dietary habit and managing diabetes |
| **Module 7** | Behavior change: Alcohol consumption, smoking |
| **Module 8** | Foot care for management of diabetes |
| **Module 9** | Diabetes and oral health |
| **Module 10** | Sexual health and diaebtes |
| **Module 11** | Travel tips and sick days |
| **Module 12** | Social and emotional supports/ meeting with health professionals |
